# Quality of Neonatal Care: A Health Facility Assessment in Balochistan Province, Pakistan

**DOI:** 10.7759/cureus.22744

**Published:** 2022-03-01

**Authors:** Vikram Kumar, Bibi Shazia Ali, Erum Choudry, Sheharyar Khan, Kamran Baig, Naveed Ur Rehman Durrani, Syed Rehan Ali

**Affiliations:** 1 Neonatology, Indus Hospital and Health Network, Karachi, PAK; 2 Paediatrics and Child Health, The Aga Khan Hospital, Karachi, PAK; 3 Indus Hospital Research Center, Indus Hospital and Health Network, Karachi, PAK; 4 Family Medicine, Baqai Medical University, Karachi, PAK; 5 Public Health, Institute of Infection Prevention & Research, Karachi, PAK; 6 Neonatology, Sidra Medicine & Weill Cornell Medicine, Doha, QAT

**Keywords:** pakistan, balochistan, quality patient care, neonatal health, health facility

## Abstract

Introduction

Balochistan is the largest of Pakistan's four provinces, yet it is also the poorest and most impoverished, particularly in terms of neonatal healthcare. In order to build and tailor strategies to improve neonatal outcomes, it is necessary to identify barriers and facilitators for interventions. Therefore, we conducted this study to provide an overview of neonatal healthcare quality and assess the structural capacity for the improvement and further development of neonatal healthcare facilities in Balochistan.

Methods

A descriptive, observational, cross-sectional study was conducted in Balochistan, a province of Pakistan. The survey was designed to assess the level of staffing and facilities in the neonatal health care units. Data were gathered through trained staff either by in-person visits to the facility or via telephone.

Results

A total of 177 facilities were assessed in 25 districts of Balochistan. A majority (88.7%) of the facilities were from the public sector. Birth and neonatal care services were provided at only 63 (36%) of the assessed facilities and only three had newborn intensive care units (NICUs) with a 1:5 staff: patient ratio. Unfortunately, all NICUs lacked the basic advanced facilities. None of the hospitals had an infection control policy or staff nor any training program for doctors.

Conclusion

In conclusion, healthcare facilities to manage neonatal patients requiring hospital care are extremely limited in Balochistan and the ones that are available have very limited resources. To improve the healthcare system in Balochistan, all stakeholders should be involved in the planning, decision-making, and implementation of healthcare programs at all levels to ensure sustainability and efficiency.

## Introduction

According to the United Nations Children's Fund (UNICEF), at least 130 million infants are born each year, of which approximately 2.4 million children die within the first 28 days of life, accounting for roughly half of all child deaths under the age of five [1-2]. More than 90% of these fatalities occur in developing countries, primarily in Sub-Saharan Africa and South Asia, whereas in 2019, the neonatal mortality rate was estimated at 25-27 deaths per 1,000 live births [1,3]. An infant born in a developing country is 10 times more likely to die in the first month than an infant born in a developed country [4]. Pakistan ranks third among these developing countries with an estimated 298 000 neonatal deaths per year and a neonatal mortality rate (NMR) of 42 deaths per 1000 live births annually [5-6]. Pakistan accounts for 7% of neonatal deaths globally, with birth asphyxia (23%), infection (36%), and preterm birth (28%) as prime causes, all of which are preventable [5]. In Pakistan, marked disparities exist in the chances of survival for infants based on their provinces, regions (urban or rural), and district of birth. Among the four provinces of Pakistan, Balochistan is the largest but the poorest and most deprived of all. Despite being the least populated, it has the highest burden of neonatal mortality, i.e., 63 deaths per 1000 live births [7]. Surprisingly, this rate is comparable to Punjab, one of the most populous provinces in the country. The most prominent factors contributing to the alarming neonatal mortality rate are lack of postnatal care for infants within 48 hours, poor maternal breastfeeding habits, and immunization follow-ups. Other causes include premature birth complications, intrapartum complications, pneumonia, diarrhea, neonatal sepsis, and infection [8]. According to the Pakistan Department of Health Survey (DHS) of 2017-18, only 34.3% of newborns in Balochistan receive postnatal care during the first 48 hours compared to Punjab and Sindh, where 69% and 75.1% of newborns receive this facility. Regional variation also indicates that all basic vaccination coverage is least prevalent among children of Balochistan (29%) versus Punjab (80%), with the highest, followed by Khyber Pakhtunkhwa (55%), and Sindh (49%) [6].

Among the many factors contributing to Balochistan's devasted healthcare system, the three most crucial deficiencies are: 1) lack of birth centers, 2) scarcity of skilled birth attendants, and 3) inadequacy of well-equipped healthcare facilities [9]. Implementation of simple, effective, and affordable interventions and improved primary obstetric and neonatal care can help improve healthcare delivery in Balochistan. To prioritize the health care needs and appropriate resource allocations, and formulate a national policy, it is necessary to address the significant evidence gap in the quality of newborn facility care. In order to accomplish this, we must have assessment data about health care facilities, disease burden, resource availability, and training in basic resuscitation skills. Therefore, we conducted this study to provide an overview of neonatal healthcare quality and assess the structural capacity for quality of newborn care in health facilities in Balochistan. We anticipated that identifying the accessibility and utilization issues to neonatal health in Balochistan could guide decision-makers to take the required measures towards appropriate and equitable health care.

## Materials and methods

A descriptive observational cross-sectional study was conducted from May 2020 to September 2020 after obtaining ethical approval (IRD_IRB_2019_10_002) from the Institutional Review Board (IRB) of the Indus Hospital and Health Network, Karachi. The survey questionnaire was prepared in consultation with neonatologists, nurses, epidemiologists, and public health professionals from various institutes. The survey was created to assess the level of staffing and facilities in the neonatal health care units of Pakistan's Balochistan province. It also covered the current state of human resources involved in newborn care and their level of training, the availability of life-saving equipment, medications, and the overall environmental conditions at all healthcare facilities (primary, secondary, and tertiary) in Balochistan, including both public and private sector hospitals. Informed consent was obtained from all participants before administering the survey, and participants were also consented to the results to be published later.

Study data were collected and managed using an online Google forms tool hosted at the department of pediatrics and neonatal division of Indus Hospital and Health Network, Karachi. A co-investigator from Balochistan province was responsible for data collection. To avoid data duplication and ensure authenticity, the survey was sent out through email to senior faculty members at the studied health facility. Data were also gathered via telephone and, training was provided to five post-graduate trainees by the primary investigator to ensure consistency in responses. Adequate time was given to each respondent to complete the questionnaire or to answer survey questions appropriately.

Data were analyzed using Statistical Package for Social Sciences (SPSS) Version 25 (IBM Corp., Armonk, NY). Frequency and percentages were calculated for each categorical variable and presented in tables and figures where appropriate.

## Results

A total of 177 facilities were assessed in 25 districts of Balochistan. A majority (88.7%) of the facilities were from the public sector, followed by the private sector (8.5%) and semi-private and charity hospitals (2.8%). Table 1 shows the characteristics of available healthcare facilities. The assessment included 145 (82%) primary health care (PHC) facilities, 27 (15.2%) secondary healthcare (SHC) facilities, and only five (2.8%) tertiary healthcare facilities in the province.

**Table 1:** Characteristics of available healthcare facilities

Characteristics	n (%)
Category of facilities	
Government	157(88.7)
Private	15(8.5)
Semi-private and charity based	5(2.8)
Level of facilities	
Primary care	145 (82)
Secondary care	27 (15.2)
Tertiary care	5 (2.8)
Number of deliveries per month	
No of deliveries	114 (64.41)
<50	25 (14.1)
50 - 100 births	11 (6.2)
More than 100 births	27 (15.2)
Labor room	
Yes	46 (26.0)
No	131 (74.0)
Operation theater	
Yes	25(14.1)
No	152 (85.6)
Well newborn nursery	
Yes	28 (15.8)
No	149 (84.2)
Special care nursery	
Yes	9 (5.1)
No	168 (94.9)
NICU	
Yes	3 (1.7)
No	174 (98.3)
Available support facilities	
Blood bank	
Yes	3(1.7)
No	174(98.3)
Laboratory & advanced imaging (Ultrasound, MRI, CT scan) services	
Yes	2 (1.1)
No	175 (98.9)
Adult ICU	
Yes	2 (1.1)
No	175 (98.9)
Dedicated emergency department	
Yes	1 (0.6)
No	176 (99.4)

Birth and neonatal care services were provided at only 63 (36%) of the assessed facilities. Almost three-fourths of the PHCs and 7% (n=2) of SHCs did not offer these services (Table 2).

**Table 2:** Availability of birth services

If the Facility Provides Birth Services	Type of Facilities							
	Primary		Secondary		Tertiary		Total	
	n	%	n	%	n	%	n	%
No	112	77.2%	2	7.4%	0	0.0%	114	64.4%
Yes	33	22.8%	25	92.5%	5	100.0%	63	35.6%
Total	145	100.0%	27	100.0%	5	100.0%	177	100.0%

Among 33 PHCs, most (n=27, 82%) facilities offered birth services through trained birth attendants while only five had trained obstetricians. Alarmingly, almost half of the facilities provided neonatal services through Lady Health Workers (LHWs). At only 11 (33%) PHCs, physicians were providing neonatal care services.

Among 25 SHCs, most (n=22, 92%) of the facilities offered birth services through trained and qualified obstetricians. At almost half of the SHCs, trained/qualified pediatricians provided neonatal care services while at 11 (41%) of the SHCs, these services were offered through GPs. In all five tertiary care facilities, trained/qualified obstetricians provided birth services while neonatal care services were provided by the trained/qualified pediatrician or neonatologist, as shown in Table 3.

**Table 3:** Service providers at neonatal healthcare facilities

Services	Providers	Type of Facilities							
		Primary		Secondary		Tertiary		Total	
		n	%	n	%	n	%	n	%
Birth services	Trained Obstetrician	5	15.2%	17	70.8%	2	66.7%	24	40.0%
	Qualified Obstetrician	0	0.0%	5	20.8%	1	33.3%	6	10.0%
	Trained Birth Attendant	27	81.8%	2	8.3%	0	0.0%	29	48.3%
	Unskilled Attendant	1	3.0%	0	0.0%	0	0.0%	1	1.7%
Total		33	100.0%	24	100.0%	3	100.0%	60	100.0%
Neonatal care services	Birth Attendant	6	18.2%	0	0.0%	0	0.0%	6	9.5%
	General Practitioner	5	15.2%	11	40.7%	0	0.0%	16	25.4%
	Lady Health Worker	16	48.5%	2	7.4%	0	0.0%	18	28.6%
	Qualified Obstetrician	0	0.0%	1	3.7%	0	0.0%	1	1.6%
	Qualified Neonatologist/Pediatrician	2	6.1%	5	18.5%	2	66.7%	9	14.3%
	Trained Obstetrician	2	6.1%	0	0.0%	0	0.0%	2	3.2%
	Trained Pediatrician	2	6.1%	8	29.6%	1	33.3%	11	17.5%
Total		33	100.0%	27	100.0%	3	100.0%	63	100.0%

Table 4 shows the available facilities and practices at NICUs. All three facilities had a staff: patient ratio of 1:5, and only about 15% of doctors and staff were trained or certified in neonatal resuscitation programs. Out of all hospitals, only three have NICUs and none of them can resuscitate neonates less than 28 weeks.

**Table 4:** Practices and available facilities at NICUs NICU: newborn intensive care unit; PDA: patent ductus arteriosus; CPAP: continuous positive airway pressure; HBB: Helping Babies Breathe; NRP: Neonatal Resuscitation Program; HBS: Helping Babies Survive; ECEB: Essential Care for Every Baby; TPN: total parenteral nutrition; IVH: intraventricular hemorrhage

Characteristics of NICU	N (%)	Characteristics of NICU	N (%)
NICU staff: patient ratio in all three NICUs	1:5	Number of NICU beds	
		< 5	1 (33.3)
		10 to 15	2 (66.7)
Practices			
Do you resuscitate preterm neonates less than 28 weeks		Use probiotics/prophylactic fluconazole	
Yes	0 (0.0)	Yes	0 (0.0)
No	3 (100.0)	No	3 (100.0)
Percentage of staff trained/certified for neonatal resuscitation (HBB/NRP/HBS/ECEB or other)		Percentage of doctors trained/certified for neonatal resuscitation (HBB/NRP/HBS/ECEB or other)	
<10%	1 (33.3)	<10%	1 (33.3)
10-20%	0 (0.0)	10-20%	1 (33.3)
None	2 (66.7)	None	1 (33.3)
Use of aminophylline for apnea		Use of TPN	
Yes	2 (66.7)	Yes	1 (33.3)
No	1 (33.3)	No	2 (66.7)
Use of caffeine for apnea		Use of surfactant	
Yes	0 (0.0)	Yes	1 (33.3)
No	3 (100.0)	No	2 (66.7)
Use of ibuprofen/paracetamol as first-line for PDA		Medications given via infusion/syringe pump/s	
Yes	1 (33.3)	Yes	2 (66.7)
No	2 (66.7)	No	1 (33.3)
Available facilities			
Echocardiography services		Units with CPAP/ventilators/patient monitors	
Yes	2 (66.7)	Yes	2 (66.7)
No	1 (33.3)	No	1 (33.3)
Transillumination light for pneumothorax		Units to screen red reflex and hearing ability	
Yes	0 (0.0)	Yes	2 (66.7)
No	3 (100.0)	No	1 (33.3)
Ability to perform an exchange transfusion for hyperbilirubinemia		Ultrasound head for IVH screening or ROP (Retinopathy of prematurity) screening facility	
Yes	2 (66.7)	Yes	0 (0.0)
No	1 (33.3)	No	3 (100.0)
Umbilical arterial line/PICC line insertion		Umbilical venous line insertion	
Yes	0 (0.0)	Yes	3 (100.0)
No	3 (100.0)	No	0 (0.0)
Neonatal surgery facilities		Units with a centralized oxygen system	
Yes	3 (100.0)	Yes	3 (100.0)
No	0 (0.0)	No	0 (0.0)
Does your hospital/unit have an infection control department?		Does your hospital have a training program for doctors (Pediatrics/Neonatology)	
Yes	0 (0.0)	Yes	0 (0.0)
No	3 (100.0)	No	3 (100.0)

Only two (66.66%) NICUs use aminophylline while none use caffeine, prophylactic fluconazole, and probiotics for babies weighing less than 1000 grams. Two out of three NICUs had echocardiography, could medically treat patent ductus arteriosus (PDA), and had the ability of surfactant administration. Unfortunately, all NICUs lacked the ability to employ transilluminator technology to rule out pneumothorax on chest X-rays or to use the ultrasound head for intraventricular hemorrhage (IVH) or retinopathy of prematurity (ROP) screening. Two (66.7%) of the NICU had units with continuous positive airway pressure (CPAP), ventilators, and patient monitors. However, none of the hospitals had an infection control policy or staff nor any training programs for doctors.

## Discussion

Access and uptake of quality essential maternal, newborn, and child healthcare services constitute a significant development indicator [10]. Pakistan is one of the developing countries where access to health care facilities and services has been a continuing challenge in improving health indicators. Unfortunately, the unavailability of services and poor quality of care further contribute to the high poor newborn and child health outcomes in Pakistan. According to UNICEF, despite significant improvements over the few years, Pakistan ranks near the bottom among other countries when it comes to neonatal and infant mortality [11]. Marked disparities exist between healthcare facilities among different provinces and regions in Pakistan, particularly in the province of Balochistan, which lags far behind other provinces in terms of health care facilities [7]. Balochistan is the largest and least developed province situated in Pakistan's arid mountainous south-western quadrant. Compared to other regions, the rate of urbanization in this area has been slow over the last six decades, and the majority of the population is uneducated and still adheres to their traditional tribal system [12]. Balochistan’s healthcare system is confronted with a number of challenges, including a deteriorating healthcare infrastructure, a scarcity of human resources, and a lack of medicines, supplies, and equipment [13]. To our knowledge, this is the first cross-sectional survey assessing the technical and infrastructural capacities of healthcare facilities in Balochistan. Our survey revealed that healthcare facilities in Balochistan lack basic preventative and primary care neonatal health care services. Lack of trained human resources, poor infrastructure, and limited equipment and supplies were repeatedly identified at most facilities. Among the four provincial jurisdictions of Pakistan, around 71% of the country's poverty-stricken population resides in Balochistan [14]. However, its dilapidated healthcare system has made the living situation for its people more despondent [7]. Out of 177 health facilities in our study, 88.7% and 8.5% were public and private sector hospitals, respectively, which heavily lack healthcare facilities and staff. Besides, there were only five tertiary care hospitals (2.8%) in the province. Data indicated that around 30-32% of the population lack access to basic healthcare facilities, which is an alarming situation [14].

Our study found that most of the facilities have no birth services, 26% have labor rooms, and only 14% have operation theatres. According to Pakistan Maternal Mortality Survey, Balochistan has the highest maternal death, with 298 deaths per 100,000 live births [15]. The most common causes of perinatal maternal mortality are excessive bleeding, infections, and hypertensive disorders. Neonatal mortality during labor is caused by preterm labor, asphyxia, or hypoxia [16]. It is well known that complications that cause maternal death and illness also cause the greatest burden of neonatal mortality and morbidity [17]. Every pregnancy is at risk for complications, the majority of which can be successfully managed if identified and addressed on time. However, the fact that only 16% of births in Balochistan take place in health facilities presents a unique challenge [7]. The majority of factors listed above can be avoided with proper labor and birth management, which includes monitoring for prolonged obstructed labor and fetal distress [17].

Our study highlighted that only nine special care nurseries were able to care for sick stable neonates and three units could provide neonatal intensive care. Due to the limited number of NICUs, accessibility is difficult, resulting in delayed presentation, thus increasing the chances of complications and poor outcomes. The lack of essential neonatal health facilities in Balochistan may be one of the reasons for the high neonatal and infant mortality rates. To ensure that all neonates survive and thrive, there is a clear need to increase the availability of designated neonatal health facilities in Balochistan that provide basic intensive care interventions to acutely ill infants, thus, significantly improving their survival rates [18].

Knowledge and understanding of the current situation of neonatal health facilities is the first step for the future planning, development, or expansion of neonatal health facilities in Balochistan. Our survey also provides an insight into the NICU structure, staffing, and available diagnostic facilities. The availability of advanced equipment with appropriate technical support is essential for improving the outcomes in critically ill neonates [19]. It was discouraging to learn that most facilities lacked basic intensive care equipment. The picture was even bleaker when it came to advanced equipment required for complex disease management. Only two NICUS were capable of caring for neonates who needed advanced therapy such as exchange transfusions, ventilators, or CPAP support. Our survey also demonstrated that apart from inadequate available facilities, equipment, limited NICU beds, and poor staffing of these units were a major issue. Only 15% of staff had any special training in neonatal resuscitation. Available NICUs had a nurse-to-bed ratio of 1:5, and these numbers are substantially low when compared to the guidelines developed by the American Academy of Pediatrics (AAP), and the Association of Women's Health, Obstetric and Neonatal Nurses. For the lowest risk babies, they recommend a nurse-to-patient ratio of one nurse for every three to four infants, and for the most complex cases, a ratio of more than one nurse per baby [20]. Furthermore, several studies have found that understaffing not only compromises neonatal quality care but is also associated with higher rates of nosocomial infection and neonatal mortality [20-21]. There is a desperate need for academic NICU programs to address this issue in Balochistan. In Sustainable Development Goal 3, the United Nations (UN) proposes to reduce preventable newborn deaths from 22 deaths per 1,000 live births to 12 deaths per 1,000 live births by 2030 [22]. Pakistan faces enormous challenges in meeting its international obligations and agreed Millennium Development Goal targets for maternal, neonatal, and child mortality reduction [23]. The current situation of neonatal mortality in Balochistan is far behind UN targets [7]. To close this gap and meet the target, the provincial government should work with the federal government to launch various neonatal and child healthcare initiatives for advancing knowledge and skill of healthcare providers through training healthcare professionals, provision of basic equipment and drugs, and community education about antenatal and postnatal care through various campaigns. Our study has several limitations, one of which is that the data in this survey was self-reported by physicians and nurses, and like any other self-reported assessments, this limits the validity of the findings. A physical survey that may have provided a more realistic understanding of the situation was restricted due to the coronavirus disease 2019 (COVID-19) pandemic.

Recommendations

The following are the key recommendations to improve neonatal healthcare services in Balochistan: 1. Annual assessments are required at all levels of health care, from the community setting to level III referral hospitals; 2. Task sharing with mid-level care providers, such as CMWs, LHVs, and nurses, for providing maternal and newborn care services at PHCs; 3. Establishing a referral network between the community and the facility and facility to facility for the timely referral of high-risk cases to tertiary care facilities; 4. Empowering Lady Health Workers by strengthening their capacity on providing basic primary care and preventative services; 5. Other important steps that can be taken to improve Balochistan's healthcare system include raising literacy rates, increasing health budgets, reducing corruption in public health projects, regionalizing healthcare services, and promoting health education.

## Conclusions

In conclusion, healthcare facilities to manage neonatal patients requiring hospital care are extremely limited in Balochistan, and the available ones have very limited resources. To reduce preventable maternal and newborn morbidity and mortality in Balochistan, every pregnant mother and neonate should receive quality care throughout pregnancy. Good-quality care requires appropriate healthcare infrastructure, human resources, optimum expertise, skills, and capacity to deal with both stable and critical neonates, as well as complications that require timely interventions and a positive attitude of health providers. To improve the healthcare delivery system in Balochistan, all stakeholders should be involved in the planning, decision-making, and implementation of healthcare programs at all levels to ensure sustainability and efficiency.

## References

[1] (2022). Neonatal mortality. https://data.unicef.org/topic/child-survival/neonatal-mortality/.

[2] World Health Organization. (‎2006 (2022). Neonatal and perinatal mortality: country, regional and global estimates. https://apps.who.int/iris/handle/10665/43444.

[3] Lawn J.E, Cousens S, Bhutta ZA (364). Why are 4 million newborn babies dying each year?. Lancet.

[4] (2022). Newborns: improving survival and well-being. https://www.who.int/news-room/fact-sheets/detail/newborns-reducing-mortality.

[5] Sarwer A, Javed B, Soto EB, Mashwani ZU (2020). Impact of the COVID-19 pandemic on maternal health services in Pakistan. Int J Health Plann Manage.

[6] (2022). National Institute of Population Studies - NIPS/Pakistan, ICF. Pakistan demographic and health survey 2017-18. Islamabad, Pakistan. https://dhsprogram.com/publications/publication-fr354-dhs-final-reports.cfm.

[7] (2017). UNICEF Pakistan. The Situation Analysis of Children in Pakistan. The state of child rights in Pakistan. https://www.unicef.org/pakistan/reports/situation-analysis-children-pakistan.

[8] Voice of Balochistan (2022). Child mortality in Balochistan. https://voiceofbalochistan.pk/opinions-and-articles/healtheducation/child-mortality-in-balochistan/.

[9] (2022). Data collection survey on health facilities and equipment in the Islamic Republic of Pakistan: final report. -- Japan International Cooperation Agency: International Techno Center Co., Ltd., 2018.10. https://openjicareport.jica.go.jp/980/980/980_117_12322293.html.

[10] Jabeen T (2001). Health facilities in Balochistan. J Appl Sci.

[11] (2022). The poor state of Pakistan’s healthcare system. Dawn's special coverage of the Sustainable Development Goals 2015-2030 continues. https://www.dawn.com/news/1285181/the-poor-state-of-pakistans-healthcare-system.

[12] (2022). Focus on lack of development in Balochistan. https://www.thenewhumanitarian.org/fr/node/197022.

[13] Green A, Ali B, Naeem A, Ross D (2000). Resource allocation and budgetary mechanisms for decentralized health systems: experiences from Balochistan, Pakistan. Bull World Health Organ.

[14] Balochistan Drought Needs Assessment (BDNA (2022). Balochistan Drought Needs Assessment (BDNA) report February 2019. https://www.humanitarianresponse.info/en/operations/pakistan/document/balochistan-drought-needs-assessment-bdna-report-february-2019.

[15] (2022). Healthcare a luxury for people in Balochistan. https://www.geo.tv/latest/189971-only-30-to-32-per-cent-of-balochistan-have-access-to-medical-facilities.

[16] (2022). Maternal mortality. https://www.dawn.com/news/1576178/maternal-mortality.

[17] Jehan I, Harris H, Salat S (2009). Neonatal mortality, risk factors and causes: a prospective population-based cohort study in urban Pakistan. Bull World Health Organ.

[18] Institute of Medicine (US) Committee on Improving Birth Outcomes (2003). Improving Birth Outcomes: Meeting the Challenge in the Developing World. National Academies Press.

[19] Ladak LA, Hamid MH, Mirza S, Siddiqui NR, Bhutta ZA (2014). A national survey of pediatric intensive care units in Pakistan. J Crit Care Med.

[20] Rogowski JA, Staiger D, Patrick T, Horbar J, Kenny M, Lake ET (2013). Nurse staffing and NICU infection rates. JAMA Pediatr.

[21] Sherenian M, Profit J, Schmidt B, Suh S, Xiao R, Zupancic JA, DeMauro SB (2013). Nurse-to-patient ratios and neonatal outcomes: a brief systematic review. Neonatology.

[22] United Nations (2022). Sustainable development goals report 2020. Report.

[23] Bhutta ZA, Hafeez A (2015). What can Pakistan do to address maternal and child health over the next decade?. Health Res Policy Syst.

